# Identification of a core EMT signature that separates basal-like breast cancers into partial- and post-EMT subtypes

**DOI:** 10.3389/fonc.2023.1249895

**Published:** 2023-12-04

**Authors:** Erik Knutsen, Saikat Das Sajib, Tonje Fiskaa, James Lorens, Thorarinn Gudjonsson, Gunhild M. Mælandsmo, Steinar Daae Johansen, Ole-Morten Seternes, Maria Perander

**Affiliations:** ^1^ Department of Medical Biology, Faculty of Health Sciences, UiT the Arctic University of Norway, Tromsø, Norway; ^2^ Centre for Clinical Research and Education, University Hospital of North Norway, Tromsø, Norway; ^3^ Department of Biomedicine, University of Bergen, Bergen, Norway; ^4^ Department of Anatomy, Faculty of Medicine, School of Health Sciences, University of Iceland, Reykjavik, Iceland; ^5^ Department of Hematology, Landspitali, University Hospital, Reykjavik, Iceland; ^6^ Department of Tumor Biology, Institute for Cancer Research, Oslo University Hospital, Oslo, Norway; ^7^ Genomics Division, Faculty of Bioscience and Aquaculture, Nord University, Bodø, Norway; ^8^ Department of Pharmacy, Faculty of Health Sciences, UiT The Arctic University of Norway, Tromsø, Norway

**Keywords:** epithelial-mesenchymal transition, gene expression signature, breast cancer, RNA-Seq, EMT-transcription factors

## Abstract

Epithelial-mesenchymal transition (EMT) is a cellular plasticity program critical for embryonic development and tissue regeneration, and aberrant EMT is associated with disease including cancer. The high degree of plasticity in the mammary epithelium is reflected in extensive heterogeneity among breast cancers. Here, we have analyzed RNA-sequencing data from three different mammary epithelial cell line-derived EMT models and identified a robust mammary EMT gene expression signature that separates breast cancers into distinct subgroups. Most strikingly, the basal-like breast cancers form two subgroups displaying partial-EMT and post-EMT gene expression patterns. We present evidence that key EMT-associated transcription factors play distinct roles at different stages of EMT in mammary epithelial cells.

## 1 Introduction

Epithelial-mesenchymal transition (EMT) is a cellular transdifferentiation process that converts epithelial cells to mesenchymal-like cells with migratory and invasive capabilities (1–4). During EMT, the epithelial cells lose their polarized organization and cell-cell junctions and undergo changes in the cytoskeleton architecture that alter the cell morphology. EMT is crucial for developmental processes like gastrulation, neural crest formation, and organogenesis, and for wound healing and regeneration of adult tissues (5). Importantly, EMT is associated with pathological conditions like cancer and fibrosis (5–7). EMT is driven by gene expression reprogramming, which is orchestrated by a group of transcription factors (TFs) commonly referred to as EMT TFs, including the SNAIL family (SNAI1/SNAIL and SNAI2/SLUG), the zinc finger E-box binding homeobox (ZEB) family (ZEB1 and ZEB2), and the Twist family BHLH transcription factors (TWIST1 and TWIST2) (2, 4, 8). A key event in EMT is transcriptional repression of the E-cadherin protein encoded by the *CDH1* gene, which is conferred by direct binding of EMT TFs to E-box motives within the *CDH1* promoter (9). This is accompanied by transcriptional upregulation of a range of genes required for the acquisition of mesenchymal traits (3, 4, 8). Historically, EMT and opposite mesenchymal-epithelial transition (MET), were thought to operate as binary switches regulating the transition between two well-defined cellular phenotypes. This notion has now changed as it has become clear that EMT is a dynamic process that gives rise to a range of intermediate cell states in which cells exhibit a mixture of epithelial and mesenchymal features (2, 10). Importantly, epithelial-mesenchymal plasticity contributes to heterogeneity within tumors and is associated with dissemination, invasion, and metastasis to distant organs, and therapy resistance (6, 7).

Depending on tissue and cell type, different extracellular cues activating a wide range of signaling pathways can induce EMT (4). Among them, the canonical TGF-β-SMAD signaling pathway has a prominent role in eliciting EMT in developmental processes and cancer (11). Importantly, EMT-signaling pathways and EMT TFs confer stem-cell like properties on epithelial cells, and the link between EMT and stem cells is experimentally well-established, particularly for normal and neoplastic mammary cells (2, 12–16). The mammary epithelium has a bilayered organization composed of two major epithelial lineages including the luminal epithelial cells that line the ducts, and an outer layer of contractile myoepithelial cells that face the basement membrane (17, 18). A striking feature of the mammary gland is that most of its development occurs postnatally. In line with this, the mammary epithelium is interspersed by mammary stem cells that are primarily confined to the outer basal epithelial layer (17). Both SLUG and ZEB1 are expressed in mammary stem cells and have been demonstrated to be important for their stem cell state (12, 14–16).

There is a growing awareness that EMT gives rise to a range of cellular states determined by extracellular cues and pre-existing differentiation states of the cell (2). To study the EMT program in mammary epithelial cells, we employed three well-defined mammary epithelial EMT cell models comprising epithelial and post-EMT derivatives of MCF10A, HMLE, and D492 cells. These models, established to represent normal breast epithelial cells, display differences reflecting their isolation and immortalized strategies. The MCF10A cell line is a spontaneously immortalized derivative of cells originally isolated from proliferative human fibrocystic mammary tissue that undergo EMT upon TGF-β treatment (19, 20). The HMLE cell line was originally isolated from reduction mammoplasty tissue samples and immortalized by expression of human telomerase reverse transcriptase (hTERT) and SV40 large-T antigen (21). HMLE cells most likely derive from multipotent mammary stem cells and naturally give rise to a cell subpopulation that displays stem cell- and post-EMT characteristics (12). D492 cells are a suprabasal-derived EpCAM-positive/sialomucin (MUC)-negative population with stem cell-like properties that were originally isolated from reduction mammoplasties (22). The cells were immortalized by expression of human papilloma virus (HPV)-16 E6 and E7 genes. D492 cells undergo EMT upon 3D cocultivation with endothelial cells (22, 23).

To identify a global mammary EMT-gene expression signature, we analyzed whole-transcriptome data from epithelial and post-EMT populations of HMLE, MCF10A, and D492 cells. We demonstrate that a 265-gene mammary EMT signature is highly robust in distinguishing breast cancer cell line subtypes and that it separates breast cancers into distinct subgroups. Most strikingly, the 265-gene mammary EMT signature separates basal-like breast cancers into two subgroups displaying either post-EMT or partial-EMT gene expression features. Finally, we present evidence that ZEB1 plays a critical role in upregulation of gene expression during EMT and is required for mammary epithelial cells to undergo complete EMT.

## 2 Materials and methods

### 2.1 Cell culturing

MCF10A cells were purchased from the American Type Culture Collection (ATCC). HMLE cells were a kind gift from Robert Weinberg, Whitehead Institute for Biomedical Research and Department of Biology, Massachusetts Institute of Technology. D492 cells were generated as previously described (22). MCF10A cells were cultured in DMEM/F12 (ThermoFisher Scientific), supplemented with 5% horse serum (ThermoFisher Scientific), 20 ng/ml EGF (R&B Systems), 0.5 μg/ml hydrocortisone (Sigma-Aldrich), 100 ng/ml cholera toxin (Sigma-Aldrich), and 10 μg/ml insulin (Sigma-Aldrich). HMLE cells were grown in a 1:1 mixture of MEBM (Lonza) with DMEM/F12 (Sigma-Aldrich) supplemented with 10 ng/ml EGF, 0.5 μg/ml hydrocortisone, 0.01 mg/ml insulin, and 1% penicillin-streptomycin. D492 cells were maintained in DMEM/F12 (Gibco), supplemented with 10 ng/ml EGF (Peprotech), 0.5 μg/ml hydrocortisone (Sigma-Aldrich), 0.25 μg/ml insulin (Sigma-Aldrich), 10 μg/ml transferrin (Sigma-Aldrich), 2.6 ng/ml sodium selenite (BD Biosciences), 10^-10^ M estradiol (Sigma-Aldrich), and 7.1 μg/ml prolactin (Sigma-Aldrich) in tissue culture treated, collagen I (Advanced Biomatrix)-coated T25 Falcon flasks (BD Biosciences). All cell lines were incubated in a 5% CO2 humidified incubator at 37°C.

### 2.2 Generation of epithelial and mesenchymal cell subpopulations for RNA-sequencing

**Figure 1:**
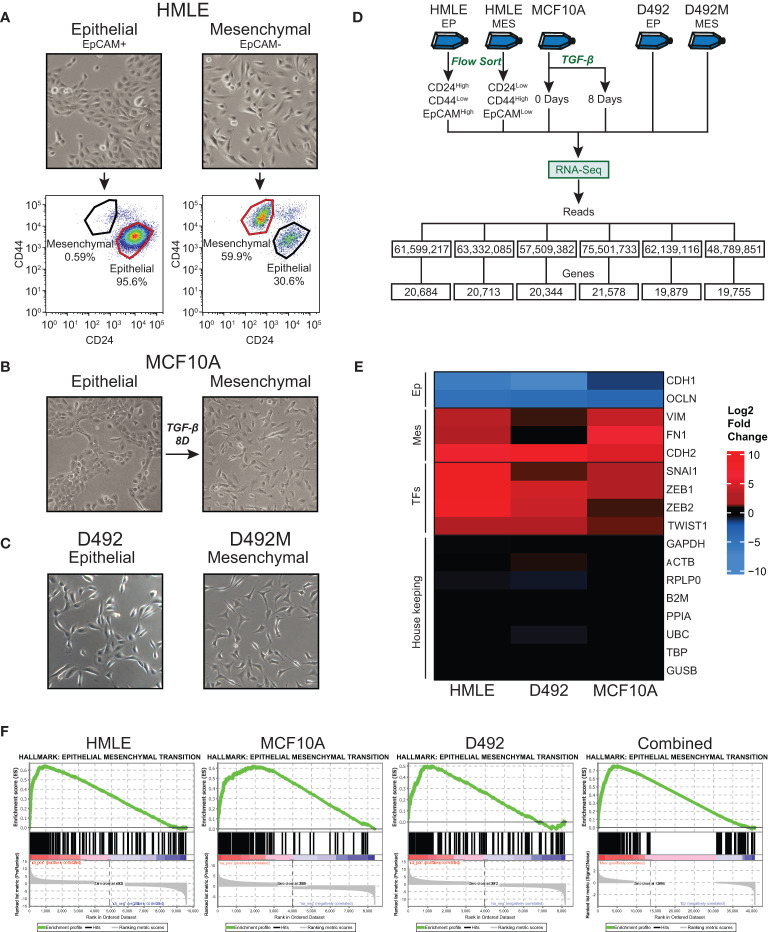
Generation and RNA-sequencing of epithelial and mesenchymal (post-EMT) subpopulations of mammary epithelial cell-derived EMT models. **(A)** EpCAM-positive and EpCAM-negative subpopulations of HMLE cells were separated using anti-EpCAM-conjugated capture beads. The cell fractions were flow cytometry-sorted into an epithelial (CD24^High^/CD44^Low^/EpCAM^High^) and a mesenchymal (CD24^Low^/CD44^High^/EpCAM^Low^) population. **(B)** MCF10A cells were left untreated or treated with 10 ng/ml TGF-β for 8 days. **(C)** D492 and D492M were cultured in 2D monolayers. **(D)** Flow chart of the RNA-Sequencing experiments. **(E)** Heatmap showing the fold change (post-EMT versus epithelial) of selected EMT marker genes, EMT transcription factors, and housekeeping genes. **(F)** Positively enriched Hallmarks with the post-EMT cells identified by Gene Set Enrichment Analysis (GSEA). The data from the three cell models are analyzed separately or combined (right panel). Ep, Epithelial; Mes, Mesenchymal; TFs, Transcription factors.

HMLE cells were separated into epithelial and mesenchymal subpopulations by immunomagnetic separation. Magnetic beads (Immunomagnetic M450 Dynabeads^®^, ThermoFisher Scientific) were coated with anti-EpCAM (MOC31, IQ Products). Trypsinized cells (1 ml) were mixed with 30 μl of coated beads and incubated on a rotating rack at 4°C for 30 min. The beads with epithelial cells and cell suspension containing mesenchymal cells, were subsequently separated using a magnet rack. HMLE cells, both EpCAM-negative and EpCAM-positive subpopulations, were grown to 80% confluence before sorted by flow cytometry according to the following procedure: Single cell suspensions were diluted in cold staining buffer (PBS containing 0.5% FCS and 3% human immune globulin (Gammaguard)) and stained with fluorescently-labelled antibodies diluted according to the manufacturer’s recommendation. Antibodies used were anti-CD24 (PE Mouse anti-human CD24, 560991, BD Biosciences), anti-CD44 (FITC Mouse anti-human CD44, 555478, BD Biosciences), and anti-EpCAM (APC mouse anti-human CD326 (EpCAM), 32408, BioLegend). Following 30 min incubation at 4°C, the stained cells were spun down and resuspended in PBS and further analyzed by LSRII flow cytometer (Becton Dickinson) using BD FACSDiva™ software. The cell populations were sorted by FACS DIVA flow cytometer (Becton Dickinson), equipped with a 488nm Argon laser (Coherent) and 633nm HeNe laser (Spectra Physics), distributing cells from each population into a separate tube containing PBS. The single cell suspensions were stained with Hoechst 33258 Staining Dye Solution prior to flow analysis for sorting to exclude the dead cells from the analysis. Unstained controls were used to set the gates. A minimum of 1,000,000 events from the viable cell population were recorded for each sample. FlowJo 7.6 software was used to analyze the data. Sorted populations were defined as indicated in **Figure 1A**. To induce EMT, MCF10A cells were treated with 10 ng/ml recombinant human TGF-β for 8 days (Bio-Techne, CFQ-331134). Both untreated (epithelial subpopulation) and treated cells (mesenchymal cell population) were harvested at 80% confluency. The generation of D492 and the post-EMT derivative, D492M, is previously described (23). Both D492 and D492M were cultivated as conventional 2D cultures and harvested at 80% confluency.

### 2.3 RNA isolation

For RNA-Sequencing, cells were lysed in 1 ml TRIzol Reagent (ThermoFisher Scientific), and the lysates were incubated at 5 min before addition of 0.2 volumes of chloroform. The samples were mixed thoroughly and incubated for 20 min on ice before centrifuged at 9000 rpm for 20 min at 4°C. The water phase was transferred to a new 1.5 ml microcentrifuge tube and 1 volume of isopropanol was added. The samples were incubated at -20°C for at least 2 hours and centrifuged at 15000 g for 30 min at 4°C. The supernatant was removed, and the pellet was washed with 1 ml 80% ice-cold EtOH before centrifuged at full speed for 5 min at room temperature. EtOH was removed and the pellet was air-dried. Total RNA was resuspended in RNase free H2O.

### 2.4 RNA-sequencing and data analyses

Ribosomal RNA depleted RNA libraries were generated and sequenced on a SOLiD5500 platform (Nord University, Bodø, Norway) as described in detail previously (24). For each cell line, a single biological replicate was used to generate one RNA-Seq library. The RiboMinus™ Eukaryote Kit (ThermoFisher Scientific) was used for rRNA depletion, and the libraries were prepared according to the SOLiD™ Total RNA-Seq Kit Protocol by Thermo Fisher Scientific. Adaptor-trimmed sequencing reads were mapped to the human genome (forward specific mapping, human reference sequence GRCh38.104) using the CLC Genomic Workbench 22. Normalized gene expression values (counts per million, CPM) were log2 transformed and calculation of significantly differentially expressed genes were done in CLC. RNA-Sequencing raw reads have been deposited to SRA (PRJNA976177). Analyzed data (full gene list) can be found in **Supplementary Table 1**. Publicly available datasets were downloaded from SRA, using the SRAtoolkit.

### 2.5 Gene set enrichment analysis

Enrichment of pathways in datasets was investigated by Gene Set Enrichment Analysis (GSEA) (25). For analyzing the enrichment in individual cell lines separately, GSEApreranked was performed with statistically significant (p < 0.05) genes ranked based on their log fold changes between epithelial and mesenchymal states. Combined GSEA of both cellular states from the three cell lines were performed using counts per million (CPM) values of 60,605 genes. The enriched pathways were identified using the MSigDB Hallmark gene set. Analysis were run with default settings, with the exception of the max size for gene set exclusion, which was set to 1000 (26).

### 2.6 Hierarchical clustering

Hierarchical clustering were performed and visualized using the R package “Complex Heatmap” to identify patterns between expression of genes across different datasets (27). For the three cell lines sequenced in this project, count per million (CPM) values were log2 normalized after adding 1 to the original values. RNA-sequencing data of the 48 breast cancer cell lines was downloaded from the Cancer Cell Line Encyclopedia (https://sites.broadinstitute.org/ccle/) (28). Gene expression data and correspondent subtype and clinical information for the TCGA cohort, were obtained using the R package TCGAbiolinks (29, 30), while for the METABRIC, the data was downloaded from the cBioPortal (https://www.cbioportal.org/) (31, 32). For all three datasets, in addition to the publicly downloaded RNA-Seq data, the expression values were z-score normalized before hierarchical clustering was performed.

The number of clusters to be selected for downstream analysis was optimized using the R package “clValid” (33). Gene expression data from CCLE, TCGA, and METABRIC were scaled separately and considered as input for the cluster stability validation function, along with internal validation, correlation metric, and complete method as the clustering and validation parameters. To determine the number of clusters, we investigated the average stability of varying number of clusters ranging from 2 to 10 (**Supplementary Figures 1A–C**). Compactness and connectedness of clusters as a measurement of stability was considered. Dunn index, silhouette width, and connectivity are three cluster stability measurements that has been widely used in biological studies for such purposes (34–36). The connectivity quantifies the extent to which datapoints are placed in the same clusters with their neighbors and therefore low connectivity indicates more stable clusters. Dunn index and silhouette width are measurements of compactness of a cluster and higher values denote more stable clusters. All three measures, in addition to information about separation of subtypes, were used for determining the optimal number of clusters for each dataset.

### 2.7 Survival, correlation, and gene copy-number analysis

Survival analyses for progression free interval (PFI) and disease specific survival (DSS) were performed by using the Kaplan-Meier method implemented in the R package “Survival” (37, 38).

Association of *ZEB1* and *SNAI1* towards the EMT states was analyzed by calculating their correlation with the EMT-down, partial-EMT and EMT-up genes annotated in the hierarchical clustering of the expression data from TCGA and METABRIC cohorts. Pearson correlation coefficients were calculated using the R package “psych”. The correlation coefficient ranges from -1 to 1, where the minimum and maximum limits denote strong negative and positive correlations, respectively. The significance of these correlations was tested by the Kruskal Wallis test and visualized using “ggpubr”.

To investigate the MYC copy number variance (CNV) in the basal-like breast cancer patients, CNV data from the TCGA BRCA cohort was downloaded using the Xena Browser by UCSC (39). In short, the CNV number estimates were generated by applying GISTIC2 and the TCGA Firehose Legacy pipeline and further thresholding the estimates into -2, -1, 0, 1, and 2 to denote CNV. The CNV data was subsetted to include only the basal-like patients from cluster 1 and cluster 5, and the negative, zero and positive values were annotated as deletion, normal, and amplification, respectively.

## 3 Results

### 3.1 Identification of a mammary EMT gene expression signature

**Figure 2:**
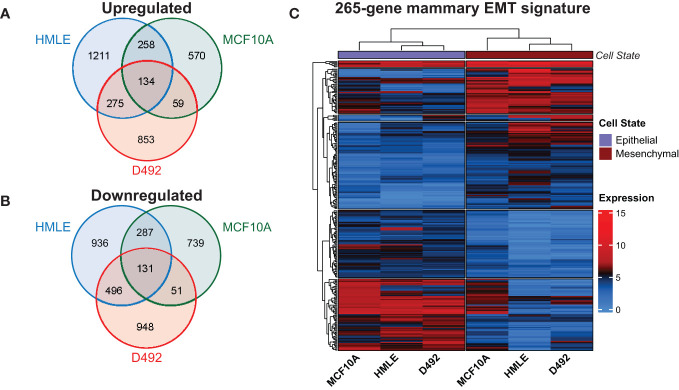
Identification of a 265-gene mammary EMT signature. **(A)** Venn diagram showing unique and shared genes that are upregulated in EMT in the three mammary epithelial cell-derived EMT models. **(B)** Venn diagram showing unique and shared genes that are downregulated in EMT in the three mammary epithelial cell-derived EMT models. **(C)** Heatmap depicting the 265 genes comprising the mammary EMT signature in the three EMT models. Expression values are log2(CPM+1). CPM: Counts Per Million.

To identify genes that change expression in EMT in mammary epithelial cells, we sequenced rRNA-depleted RNA from epithelial and post-EMT cell populations of HMLE and MCF10A. rRNA-depleted RNA from epithelial and post-EMT cell populations of D492 cells have been sequenced by us previously and the data was re-analyzed here (24). Briefly, EpCAM-positive and EpCAM-negative subpopulations of HMLE cells were obtained using anti-EpCAM-conjugated capture beads, and both cell fractions were further flow cytometry-sorted into an epithelial (CD24^High^/CD44^Low^/EpCAM^High^) and a mesenchymal (CD24^Low^/CD44^High^/EpCAM^Low^) population (**Figure 1A**). MCF10A cells were either left untreated or treated with TGF-β for 8 days to induce EMT (**Figure 1B**). Picture of D492 and its stable post-EMT derivative, D492M cultured in conventional 2D monolayers is shown in **Figure 1C**. The epithelial and mesenchymal populations of the three cell lines displayed distinct morphologies with the more elongated post-EMT cells forming fewer cell-cell contacts compared to the cognate epithelial state (**Figures 1A–C**). rRNA-depleted RNA was subjected to sequencing and an average of 61.5 +/- 8.7 million reads were generated for each cell model (**Figure 1D**). Adaptor- and quality-trimmed reads were mapped to the human genome (GRCh38) with Ensembl gene annotation (v104), including 60,605 genes (40). An average of 20,492 +/-664 genes were expressed in the three cell models (**Figure 1D**, **Supplementary Table 1**). Using fold change >2.0, FDR p-value <0.05, and difference in CPM (counts per million) >5.0 as criteria, 3,728, 2,227, and 2,945 genes were found to be differentially expressed in epithelial and post-EMT populations of HMLE, MCF10A, and D492, respectively. Importantly, genes encoding mesenchymal markers including N-cadherin (*CDH2*), vimentin (*VIM*), and fibronectin (*FN1*), as well as key EMT transcription factors including SNAIL (*SNAI1*), TWIST1, ZEB1, and ZEB2 were induced in all three EMT experiments (**Figure 1E**). Concomitantly, the expression of the epithelial genes encoding E-cadherin (*CDH1*), and occludin (*OCLN*) were downregulated (**Figure 1E**). We performed Gene Set Enrichment Analysis (GSEA) using the Gene Hallmarks for computing overlap for each of the cell lines (25, 41, 42). The input from each model system was genes with an FDR p-value <0.05, ranked according to their log2 fold changes, including 9,864, 8,688, and 8,175 genes for HMLE, MCF10A, and D492, respectively. For all three models, “Epithelial Mesenchymal Transition” was the most significantly enriched hallmark associated with the post-EMT cells (**Figure 1F**, **Supplementary Table 2**). The hallmark consists of 200 EMT-related genes, of which 80, 99, and 66 were found to be enriched in HMLE, MCF10A, and D492, respectively (**Supplementary Table 3**). Furthermore, when combining the data from the three EMT models, the EMT hallmark gene set was again the most significantly enriched, with 126 out of 200 genes defined as significant (**Figure 1F**, right panel). By comparing the significantly up- and downregulated genes across the three EMT models, we found 134 to be significantly upregulated in all three models (**Figure 2A**), and 131 to be significantly downregulated (**Figure 2B**), giving a common signature of 265 EMT-associated genes which we hereafter refer to as the “mammary EMT signature” (**Figure 2C**, **Supplementary Table 4**). We then went on to compare our signature with other signatures launched in the EMTome database, which includes 84 EMT signatures (**Supplementary Table 5**) (43). From our signature, 163 genes (62%) were found in at least two signatures, whereas 57 genes (22%) were not reported in any of the previous signatures. The 20 genes, from our signature, that were most frequently found in other EMT signatures were *VIM*, *CDH1*, *ZEB1*, *CDH2*, *ZEB2*, *SNAI1*, *CCN2*, *TWIST1*, *EPCAM*, *CLDN4*, *SERPINE1*, *ESRP1*, *WNT5A*, *ST14*, *ITGA5*, *RAB25*, *COL5A2*, *ERBB3*, *OCLN*, and *MAP7*, of which the majority has been associated with the EMT process (**Supplementary Table 5**). When only considering signatures derived from breast cancer cell lines or patient samples, disregarding signatures containing <50 genes, the average overlap between our and the other signatures was 14%. This is a higher average overlap than what is found between the existing breast cancer-related signatures within the EMTome database, where the highest average overlap for a specific signature was 11%. Together, this indicates that we have retrieved a robust mammary EMT signature.

### 3.2 Breast cancer cell line subtypes are distinguished by the mammary EMT gene expression signature

**Figure 3:**
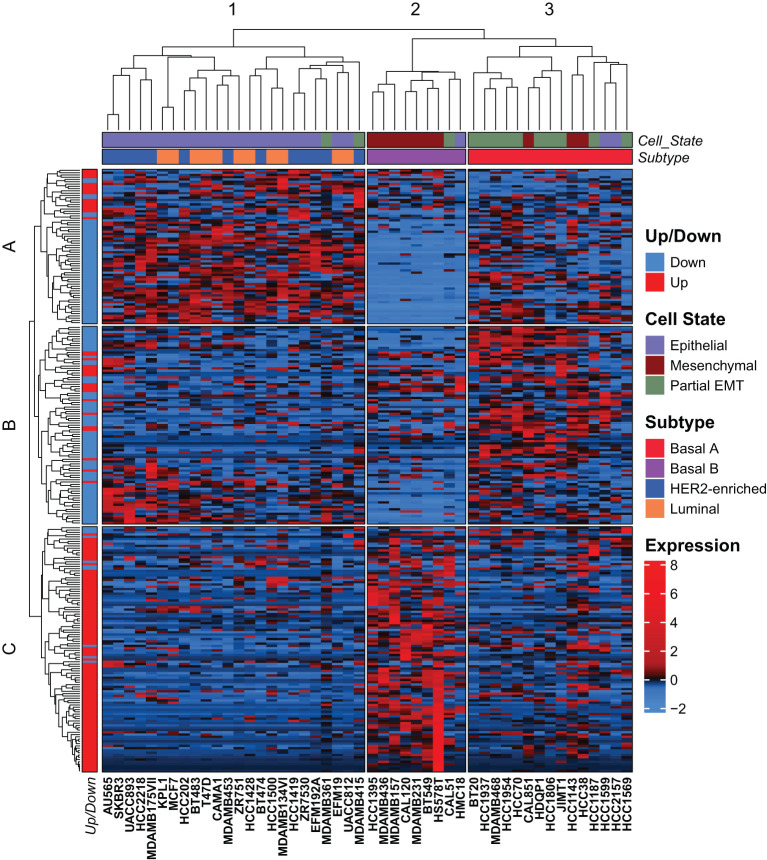
The mammary EMT signature separates breast cancer cell lines into three clusters. Hierarchical clustering of 48 breast cancer cell lines based on the mammary EMT signature. Subtype and cell state based on previous clustering are shown as top banners above the heatmap. Expression values are row-based z-normalized reads per kilobase per million (RPKM). Cell lines (columns) are clustered into the three clusters 1-3, while genes (rows) are clustered into three clusters A-C.

Breast cancer is generally characterized by high degree of both inter- and intratumor heterogeneity determined by cell type and/or differentiation state of the originating mammary cell, the acquired mutational landscape, copy-number alterations, and epigenetic mechanisms (44, 45). However, global gene expression analyses have led to the identification of gene expression signatures that stratify breast cancer into molecular subtypes. In the pioneer work by Sørlie and Perou, five intrinsic subtypes were identified, luminal A, luminal B, basal-like, HER2-enriched, and normal-like, that largely reflected the clinical classification of breast cancer subtypes based on the expression of hormone receptors, HER2, and Ki67 (46, 47). Breast cancer cell lines are frequently used in studies of breast cancer subtypes, and transcriptomic analyses of 51 breast cancer cell lines showed that they formed two major clusters identified as luminal and basal-like, of which the latter further branched into two clusters referred to as basal A and basal B (48). To determine whether the mammary EMT signature associates with specific breast cancer subtypes, we analyzed the expression of the 265-gene mammary EMT signature in 48 breast cancer cell lines from the Cancer Cell Line Encyclopedia (CCLE) that classifies the cell lines as luminal, basal A, basal B, or HER2-enriched (49). The expression data was z-normalized to prevent any bias from absolute expression, as absolute expression values are influenced by the methods used for gene expression analyses (50, 51). Two out of the 265 genes, *SIK1B* and *ENSG00000267748*, were not identified in the CCLE gene list. We performed hierarchical cluster analysis of the 263 remaining genes, which branched into three gene clusters (A-C) that divided the cell lines into three clusters (1-3) (**Figure 3**, **Supplementary Figure 1A**). The cell clusters nicely overlapped with three clusters previously identified by us using the EMT Hallmark gene set, which we identified as “Epithelial”, “Partial EMT”, and “Mesenchymal” cell states (**Figure 3**, Cell state) (52). This indicates that the genes in our mammary EMT signature, are associated with the EMT process. Moreover, the mammary EMT signature clearly separated the breast cancer cell lines according to their subtypes (**Figure 3**, Subtype) as sample cluster 2 only consisted of cells with the basal B subtype and cluster 3 only consisted of cells with the basal A subtype. As reported previously for gene expression signatures, our mammary EMT signature was not able to separate luminal and HER2-enriched cell lines that formed one distinct cluster (cluster 1) (48). We examined the genes from the three gene clusters A, B, and C (**Supplementary Table 6**) for the percentages of upregulated genes identified in our EMT models. Here, gene cluster A and B had low percentages of upregulated EMT genes, 24% and 22%, respectively (**Figure 3**, Up/Down). In sharp contrast, gene cluster C had 91% upregulated genes, meaning that this cluster is enriched for mesenchymal-associated genes (**Figure 3**, Up/Down). The genes within gene cluster C are most highly expressed in cell line cluster 2, which is constituted by basal B cell lines (**Figure 3**). The basal B cell lines have previously been shown to have similar expression pattern as claudin-low tumors, which is associated with post-EMT gene expression signatures (53, 54). Taken together, the mammary EMT signature separates breast cancer cell line subtypes in a similar manner as whole transcriptome profiles. This indicates that the EMT state is a key feature that distinguishes breast cancer cell lines.

### 3.3 The mammary EMT gene expression signature separates basal-like breast cancer into two groups

**Figure 4:**
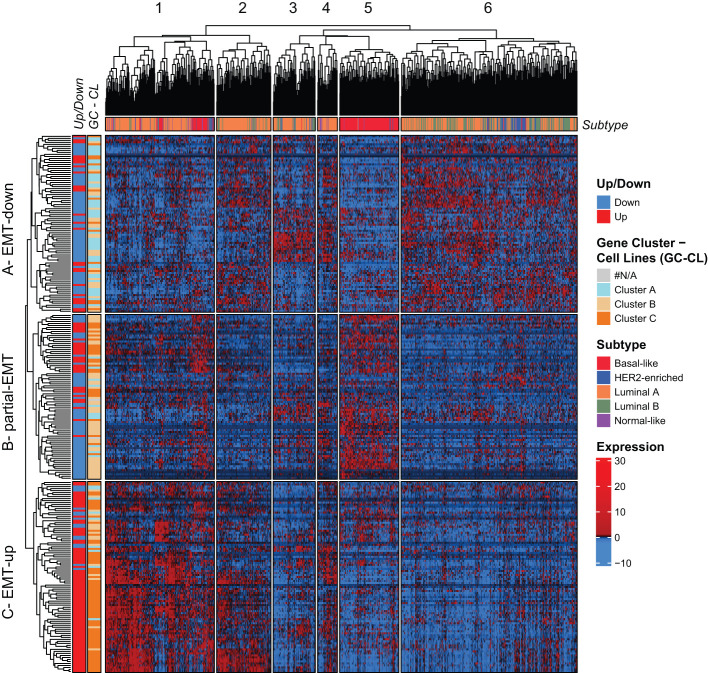
The mammary EMT signature separates breast cancers from the TCGA cohort into six subgroups. Hierarchical clustering of 1041 breast cancer patients from the TCGA cohort based on the mammary EMT signature. Subtype is shown as a top banner above the heatmap, while genes found to be up- or downregulated in the EMT cell lines models (Up/Down) and the gene clusters from the breast cancer cell lines (GC-CL) clustering are shown as side banners on the left side of the heatmap. Expression values are row-based z-normalized reads per kilobase per million (RPKM). Patients (columns) are clustered into the six clusters 1-6, while genes (rows) are clustered into three clusters A – EMT-down, B – partial-EMT, and C – EMT-up.

We went on to examine the expression of the mammary EMT signature in RNA-sequencing data from 1041 breast cancer patients from the TCGA cohort that were stratified according to the PAM50 signatures into luminal A (540 patients), luminal B (201 patients), HER2-enriched (80 patients), basal-like (182 patients), and normal-like (38 patients) subtypes (55). The mammary EMT signature again branched into three gene clusters (A-C), that separated the patients into six clusters (1-6) (**Figure 4**, **Supplementary Figure 1B**). The gene clusters displayed significant overlap with the gene clusters from the cell line analyses (72%, 52%, and 64% of genes in clusters A, B, and C, respectively) (**Figure 4**, Gene Cluster –Cell Line, **Supplementary Table 6**). Cluster A was defined by high expression of genes that were downregulated in the EMT process and the cluster was therefore renamed to “EMT-down”. Cluster B was typified by expression of genes that were either upregulated or downregulated in EMT and we named this cluster “partial-EMT”. Finally, cluster C, referred to as “EMT-up”, was mainly constituted by genes that were upregulated in EMT. This clearly indicates that the mammary EMT signature distinguishes breast cancers according to their EMT state and separates them into cancers with epithelial, partial-EMT, or post-EMT (mesenchymal) features. Of the six patient clusters, cluster 5 was clearly enriched for basal-like cancers (70% of all basal-like cancers), whereas the remaining basal-like breast cancers were within cluster 1 (**Figure 4**, Subtype). Most of the luminal B and HER2-enriched breast cancers resided within cluster 6 (74% and 64%, respectively). In contrast, luminal A cancers were scattered among cluster 1, 2, 3, and 6 (27%, 19%, 12%, and 35%, respectively). Cluster 5, that was dominated by basal-like cancers, was clearly defined by high expression of genes within the “partial-EMT” cluster. Moreover, patient cluster 1, which is constituted by a mixture of breast cancer subtypes including the remaining basal-like cancers, was defined by high expression of genes within the “EMT-up” cluster. Finally, cluster 6 was defined by high expression of genes within the “EMT-down” cluster. This indicates that the majority of luminal B and HER2-enriched breast cancers in the TCGA cohort display epithelial traits.

**Figure 5:**
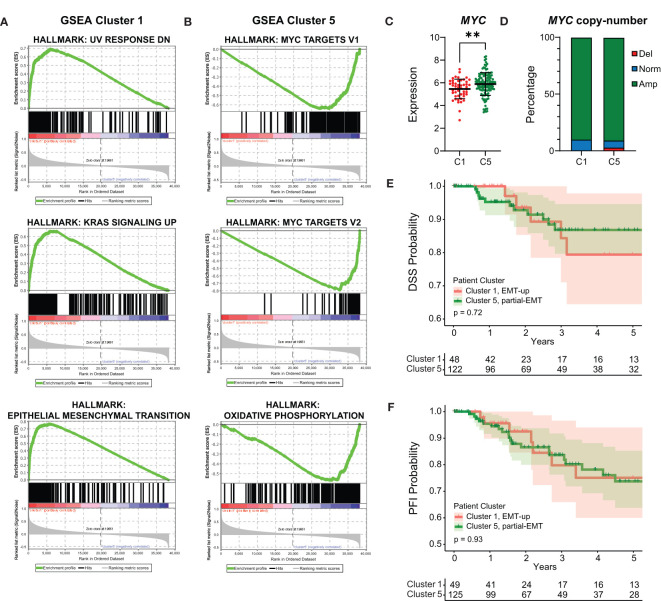
Basal-like cancers with post-EMT and partial-EMT gene expression patterns display different regulation of cellular pathways, but no evident difference in prognosis. **(A, B)** Gene Set Enrichment Analysis (GSEA) for genes that are associated with basal-like patients from post-EMT cluster 1 **(A)** and partial-EMT cluster 5 **(B)**. **(C)** *MYC* expression in TCGA post-EMT cluster 1 (C1) and partial-EMT cluster 5 (C5). Expression values are log2(RPKM+1). **(D)** *MYC* copy-number status in TCGA post-EMT cluster 1 (C1) and partial-EMT cluster 5 (C5). **(E, F)** Kaplan-Meier curve displaying the estimated Disease Specific Survival (DSS) **(E)** and Progression Free Interval (PFI) **(F)** up to five years for basal-like breast cancer patients clustered in the EMT-up cluster 1 or the partial-EMT cluster 5. RPKM, Reads per kilobase per million; Del, Deletion; Norm, Normal; Amp, Amplification. ** P<0.01.

We decided to focus our analyses on the basal-like breast cancers as they are still the most aggressive breast tumors with fewest treatment options (56). The mammary EMT signature clearly separated the basal-like breast cancers into two subgroups with either EMT-up (cluster 1, n=49) or partial-EMT (cluster 5, n=127) gene expression features. To determine whether cellular pathways are differentially regulated in the two groups, we performed GSEA including all the expressed genes. The three most enriched hallmarks associated with the basal-like breast cancers within cluster 1 are “UV-response down”, “KRAS signaling up”, and “Epithelial Mesenchymal Transition” (**Figure 5A**, **Supplementary Table 7**). This indicates that the basal-like breast cancers in cluster 1 indeed formed a subgroup based on post-EMT gene expression features. The basal-like breast cancers in cluster 5, however, displayed a very different gene expression pattern and were enriched with MYC-regulated genes (“MYC targets V1” and “-V2”), E2F-target genes, and genes associated with oxidative phosphorylation (**Figure 5B**, **Supplementary Table 7**). The clear enrichment with MYC-regulated genes suggests that even though *MYC* is not part of the mammary EMT signature, basal-like breast cancers that have gained *MYC* expression seem to be overrepresented in cluster 5. We therefore determined *MYC* expression in the two subgroups of basal-like cancers and found it to be significantly elevated in basal-like cancers within cluster 5 compared to those residing in cluster 1 (**Figure 5C**). As the MYC gene is frequently amplified in breast cancers, we analyzed copy number variations in the basal-like cancers from cluster 1 and cluster 5. However, no difference in amplification of the MYC gene in these two subgroups were identified, being 89.8% in cluster 1 and 90.5% in cluster 5 (**Figure 5D**). This indicated that elevated MYC expression in basal-like cancers in cluster 5 is caused by other mechanisms than gene amplification. As the two subgroups of basal-like cancers displayed different pathway enrichment, a relevant question is whether patients within the subgroups had different clinical outcome. However, analyses of clinical data from the TCGA cohort showed that there were no significant differences in disease specific survival (DSS), progression free interval (PFI), or percentage of patients with distant metastasis for the basal-like patients in cluster 1 and cluster 5 (**Figures 5E, F**, **Supplementary Figures 2A, B**).

**Figure 6:**
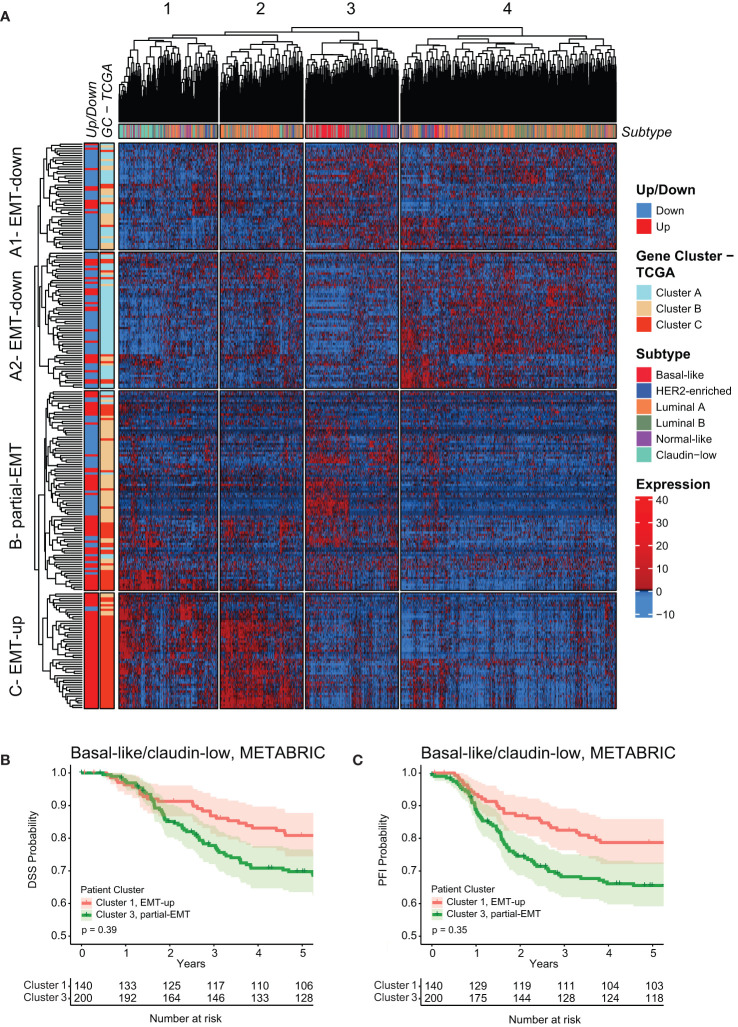
The mammary EMT signature separates breast cancers from the METABRIC cohort into four subgroups. **(A)** Hierarchical clustering of 1898 breast cancer patients from the METABRIC cohort based on the mammary EMT signature. Subtype is shown as a top banner above the heatmap, while genes found to be up- or downregulated in the EMT cell lines models (Up/Down) and the gene clusters from the TCGA (GC-TCGA) clustering are shown as side banners on the left side of the heatmap. Expression values are row-based z-normalized reads per kilobase per million (RPKM). Patients (columns) are clustered into the four clusters 1-4, while genes (rows) are clustered into four clusters A1 – EMT-down, A2 – EMT-down, B – partial-EMT, and C – EMT-up. **(B, C)** Kaplan-Meier curve displaying the estimated Disease Specific Survival (DSS) **(B)** and Progression Free Interval (PFI) **(C)** up to five years for basal-like and claudin-low breast cancer patients clustered in the EMT-up cluster 1 or the partial-EMT cluster 3.

To validate the above finding in an independent patient cohort, we applied the mammary EMT signature on microarray data from the METABRIC breast cancer cohort (n=1898), which contained expression data for 246 of the 265 genes within the signature (57). The METABRIC cohort has also stratified breast cancers as claudin-low based on a nine cell-line predictor described by Prat and co-workers (53, 54, 58). Hierarchical clustering analysis revealed that the genes of the mammary EMT signature formed an EMT-up, a partial-EMT, and two EMT-down clusters that to a large extent overlapped with the corresponding gene clusters in the TCGA cohort (**Figure 6A**, **Supplementary Table 6**). Here, the overlap was 65%, 55%, and 88% of genes in EMT-down A1 and A2, partial-EMT, and EMT-up, respectively. The mammary EMT signature separated the patients into four clusters (**Figure 6A**, **Supplementary Figure 1C**). Similarly to cancers within the TCGA cohort, the majority of the basal-like cancers formed a subgroup with a “partial-EMT” gene expression pattern (cluster 3) that was enriched with MYC target genes (**Supplementary Table 7**). Moreover, most of the claudin-low breast cancers (65%), resided in cluster 1 that, together with cluster 2, is defined by genes that are upregulated in EMT. Of note, claudin-low breast cancers have recently been shown to be overrepresented by basal-like breast cancers (58). Finally, we combined clinical data for the basal-like and claudin-low cancers within cluster 1 and 3 and found that, although not significant, patients within cluster 3 had worse outcome in terms of DDS and PFI than patients within cluster 1 (**Figures 6B, C**, **Supplementary Figures 2C, D**).

### 3.4 EMT TFs are responsible for the partial- and post-EMT states

**Figure 7:**
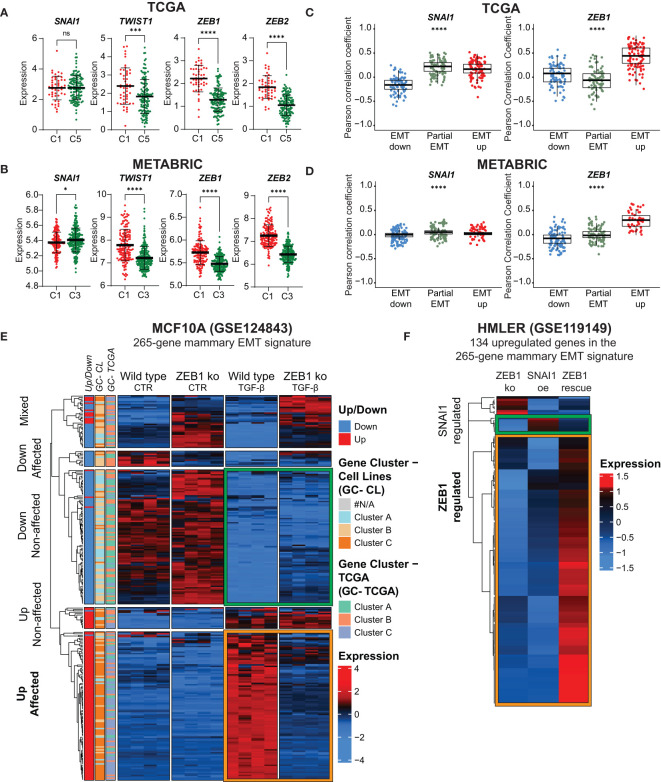
ZEB1 acts as a transcriptional activator and is required for acquisition of post-EMT gene expression pattern. **(A, B)** *SNAI1*, *TWIST1*, *ZEB1*, and *ZEB2* expression in basal-like patients from the TCGA **(A)** post-EMT cluster 1 (C1) and partial-EMT cluster 5 (C5) and METABRIC post-EMT cluster 1 (C1) and partial-EMT cluster 3 (C3). Expression values are log2(RPKM+1). **(C)** and **(D)** Correlation analysis for individual genes withing the 265-gene mammary EMT signature with *ZEB1* and *SNAI1* in TCGA **(C)** and METABRIC **(D)**. **(E)** Hierarchical clustering of the mammary EMT signature in wild type and ZEB1 knockout MCF10A cells left untreated of treated with TGF-β. Genes found to be up- or downregulated in the EMT cell lines models (Up/Down), gene clusters from the breast cancer cell lines (GC-CL) clustering, and gene clusters from the TCGA (GC-TCGA) clustering are shown as side banners on the left side of the heatmap. Expression values are row-based z-normalized counts per million (CPM). **(F)** Hierarchical clustering of the mammary EMT signature in HMLER ZEB1 knock out cells (ZEB1 ko), ZEB1 ko cells that overexpress SNAI1 (SNAI1 oe), and SNAI1-expressing cells that are rescued by ectopic expression of *ZEB1* (ZEB1 rescue). * P<0.05; *** P<0.001; **** P<0.0001. ns, not significant.

The EMT TFs play key roles in orchestrating the EMT process and their relative activities might contribute to the vast epithelial plasticity that gives rise to multiple cell states in normal physiology and cancer (3). Four genes encoding EMT TFs, *ZEB1*, *ZEB2*, *SNAI1* and *TWIST1*, are part of the generated mammary EMT signature (**Supplementary Table 4**). We postulated that these four factors have an instrumental role in forming the gene clusters that separated basal-like breast cancers into two subgroups. We therefore analyzed the partial-EMT and EMT-up gene clusters from TCGA and found that *SNAI1* and *TWIST1* were among the partial-EMT genes, whereas *ZEB1* and *ZEB2* were in the EMT-up gene cluster (**Supplementary Table 6**). Next, we plotted the expression of the four EMT TFs for the basal-like patients from cluster 1 and 5 in the TCGA (**Figure 7A**) and cluster 1 and 3 in the METABRIC cohort (**Figure 7B**). Whereas *SNAI1* was equally expressed or slightly downregulated in the partial-EMT subgroups of basal-like cancers, *ZEB1*, *ZEB2*, and *TWIST1* were more highly expressed in cluster 1 that is defined by the EMT-up genes. This suggests that the ZEB family members and/or TWIST act as transcriptional activators that play key roles in completing EMT in mammary epithelial cells that will generate cells with a highly mesenchymal phenotype. Moreover, the findings indicate that SNAIL might be instrumental for the partial-EMT gene expression signature associated with the majority of the basal-like cancers. This is in line with a recent publication showing that ZEB1 is required for complete EMT in *H-Ras^V12^* expressing HMLE cells (HMLER), whereas SNAIL is important for acquisition of a hybrid EMT state (59). To shed more light on the association between SNAIL and ZEB1 and epithelial, partial-, or post-EMT gene expression features in the TCGA and METABRIC cohort, we determined their correlation with each of the genes defining the three gene clusters (**Figures 7C, D**). Here, a strong correlation was seen between *ZEB1* and the post-EMT genes in both TCGA and METABRIC. *SNAI1*, on the contrary, showed a strong correlation for both the post-EMT genes and the partial-EMT genes in TCGA, but no specific correlation towards any of the gene sets in METABRIC. We decided to further evaluate the regulatory role of ZEB1 on our mammary EMT signature. To this end, we determined the expression of the 265 genes within the mammary EMT signature in *in silico* RNA-Seq data (GSE124843) from wild type and *ZEB1* knockout (ko) MCF10A cells that had been induced to undergo EMT by TGF-β (60). We indeed confirmed that a large majority of the genes within the mammary EMT signature followed the same expression pattern upon induction of EMT in the wild type cells in this experiment, as in our cellular models. Strikingly, 78% of the upregulated genes within the mammary EMT signature were not upregulated in the ZEB1 ko cells (**Figure 7E**, Up affected, orange square). On the other hand, the majority (71%) of the genes that were downregulated in the signature, were also downregulated in the ZEB1 ko cells (**Figure 7E**, Down non-affected, green square). To determine whether enhanced SNAI1 expression could compensate for loss of ZEB1 expression in the 134 upregulated genes within the mammary EMT signature, we assessed *in silico* RNA-seq data from HMLER cells that were forced into a stable epithelial state by knocking out ZEB1 expression (GSE119149) (59). In these cells, it has previously been shown that ectopic expression of SNAI1 drove the cells into a partial EMT state, whereas coexpression of SNAI1 and ZEB1 induced the cells to undergo complete EMT (59). Ectopic expression of SNAI1 in ZEB1 ko cells only induced the expression of a minor subset of the upregulated genes identified in the mammary EMT signature (**Figure 7F**, SNAI1 affected, green square). In sharp contrast, the majority of the genes were induced upon ZEB1 rescue (**Figure 7F**, ZEB1 affected, orange square). This clearly indicates that even though *SNAI1* is part of our mammary EMT signature, SNAIL does not mediate transcriptional upregulation of genes that is required for the EMT process to be fully completed.

## 4 Discussion

The plasticity of epithelial cells, allowing them to acquire mesenchymal traits under certain circumstances, is an essential physiological process in embryonic development and tissue regeneration. It is now generally accepted that aberrant onset of EMT is associated with serious human diseases including cancer. To provide novel insight into the EMT process in non-transformed mammary epithelial cells, we have here performed whole-transcriptome analyses of epithelial and post-EMT subpopulations of three well-defined mammary epithelial cell-derived EMT models (HMLE, MCF10A, and D492 cells). From these data, we derived a common mammary EMT signature reflecting a conserved core of 265 differentially expressed genes. Importantly, although GSEA demonstrated a clear enrichment of genes within the EMT hallmark for each of the three mammary EMT cell models, approximately 60% of the differentially expressed EMT-associated genes (57,6%, 58,7%, and 61,1% in HMLE, MCF10A, and D492, respectively), were cell type-specific. Cell type-specific changes during EMT are reflected by high degree of diversity in published EMT gene expression signatures. This is clearly seen for the EMT signatures within the EMTome database (43), in which the average overlap between any two signatures is low (≤ 11%, considering signatures consisting of >50 genes). This demonstrates the importance of including multiple cell models to identify global EMT-associated genes. The signature clearly separates established breast cancer cell lines into three groups following their subtyping as basal A, basal B, and luminal/HER2-enriched. This confirms that the EMT state is indeed a significant contributor to classification of breast cancer cell lines (48, 53, 54, 61, 62). Of note, the three cell line models used in this study display basal-like features and might therefore be better models for basal-like cancer cells. In the future, it would be interesting to see whether ER-positive normal mammary epithelial cells, like the recently obtained iHBEC^ERpos^ cell line (63), display the same level of plasticity as basal-like cells and can be included in studies aimed at better refining luminal breast cancer cell lines according to the EMT state.

We have found that the mammary EMT signature also separates breast cancers from the TCGA and METABRIC cohorts into distinct subgroups. Based on expression of the genes within the signatures, they can roughly be divided into cancers with post-EMT features (EMT-up), epithelial features (EMT-down), and partial-EMT features. With exception of luminal B and HER2-enriched cancers in the TCGA cohort, all breast cancer subtypes are represented in the post-EMT subgroup. However, tumor purity is an important issue that needs to be considered when interpreting bulk tumor gene expression data, and post-EMT features can be conferred to samples by infiltrating stromal cells (64). For instance, we noted that whereas 46% of luminal A cancers display EMT-up gene expression features, only a small subfraction of luminal B cancers have mesenchymal gene expression pattern. It is tempting to speculate that this is caused by differences in the tumor purity as luminal A tumors cancers are generally smaller than luminal B cancers, which might influence tumor cellularity in the samples (65). The fact that very few luminal breast cancer cell lines display mesenchymal characteristics, supports the notion that the post-EMT gene expression pattern seen in many luminal A breast cancer samples might be heavily influenced by non-tumor cells.

The mammary EMT signature separates basal-like breast cancers into two subgroups displaying either post-EMT (“EMT-up”) (27%, TCGA) or partial-EMT gene expression patterns (70%, TCGA). As distinct from the other breast cancer subtypes, basal-like breast cancers are overrepresented by cancers displaying partial-EMT features. Gene Set Enrichment Analyses clearly suggest that cellular pathways are differentially regulated in the two subgroups. Perhaps most strikingly, the partial-EMT gene cluster is enriched for MYC target genes, and in line with this, *MYC* expression in basal-like breast cancers with partial-EMT features is higher than in basal-like cancers with post-EMT features. Of note, *MYC* is not part of the mammary EMT signature and was not identified among the genes that changed expression in EMT in neither HMLE, D492, nor MCF10A cells. However, we can’t exclude that MYC still contributes to the partial-EMT phenotype seen in the majority of the basal-like breast cancers. The *MYC* gene is located on chromosome 8q24 that is amplified in many solid cancers (66, 67). In breast cancer, 8q24 amplification is most frequent in basal-like breast cancers and is associated with poor disease outcome (55, 57, 67–69). The enrichment with both MYC- and E2F-target genes in the basal-like breast cancers with a partial-EMT phenotype, might indicate that these cancers are highly proliferative and potentially more aggressive. Indeed, it has been demonstrated that breast cancer cells that have acquired a partial-EMT state are more tumorigenic than cancer cells that have undergone complete EMT (59, 70, 71). In the TCGA cohort, we did not find any differences in clinical outcome when comparing basal-like cancers displaying partial-EMT or post-EMT gene expression. However, in the METABRIC cohort, when considering both claudin-low and basal-like cancers, cancers with a partial-EMT phenotype display worse prognosis than cancers with mesenchymal features. This needs to be further confirmed in more cohorts.

By combining experimental and *in silico* analyses of gene expression data in mammary epithelial EMT cell models, we present evidence that SNAIL and ZEB1 play distinct roles at different states of the EMT process. Our results support previously published data showing that ZEB1 is instrumental for epithelial mammary cells to undergo complete EMT (59, 72). In line with this, we found ZEB1 to be more highly expressed in basal-like cancers displaying post-EMT gene expression patterns than in basal-like cancers displaying partial-EMT features. Moreover, our data indicate that ZEB1-regulated pathways have a particularly important role in turning on genes required for the acquisition of a mesenchymal phenotype. How ZEB1, which is thought to primarily act as a transcriptional repressor in EMT, mediates upregulation of these genes, remains to be resolved. There are indeed intricate cross-regulations between the EMT TFs, and between EMT TFs and post-transcriptional regulatory mechanisms, for instance those involving miRNAs from the miR-200 family (72). ZEB1 has been shown to function as a transcriptional activator in complex with other transcription factors, such as YAP and AP1-factors c-Jun and FOSL1 in breast cancer (73). Interestingly, we noted that *WNT5A* and *WNT5B* were among the upregulated genes in the mammary EMT signature. WNT5A/5B are ligands of the noncanonical Wnt signaling pathway, and WNT5A has previously been shown to play a critical role in maintaining HMLER cells, as well as the SUM159 breast cancer cell line, in a fully mesenchymal state (59). Noncanonical Wnt signaling activates c-Jun, and in the future it will be interesting to see whether ZEB1-cJun complexes act as transcriptional activators in mammary epithelial cells that have undergone complete EMT. For the genes within the mammary 265 gene signature, ZEB1 seems to play a less prominent role as a repressor, as 71% of the downregulated genes were still downregulated in TGF-β treated ZEB1-depleted MCF10A cells. Of note, *CDH1* was not among the ZEB1-independent genes. It is tempting to speculate that silencing of gene expression is primarily mediated by SNAIL, which has previously been shown to drive mammary epithelial cells into a partial-EMT phenotype (59). Our data is in agreement with this, as we find *SNAI1* to be part of a gene cluster that is associated with partial-EMT. Furthermore, as opposed to *ZEB1*, *SNAI1* is equally expressed in basal-like breast cancers with partial-EMT and post-EMT features. To conclude, our data support the concerted action of SNAIL and ZEB1 in mammary EMT, where the two factors have distinct roles in the transition to partial and complete EMT states.

## 5 Conclusion

To conclude, we have identified a mammary EMT signature composed of 265 gene by comparing EMT-driven changes in gene expression in HMLE, MCF10A, and D492 cells. We have demonstrated that this is indeed a powerful EMT signature and that the signature distinguishes subgroups of breast cancer tumors. Further studies will demonstrate whether the EMT signature can further stratify patients and predict the response to certain therapy regimens. Finally, we provide further evidence that SNAIL and ZEB1 play distinct roles in EMT in mammary epithelial cells.

## Data availability statement

The raw data generated from RNA sequencing has been deposited in the National Center for Biotechnology Information (NCBI) Sequence Read Archive (SRA) database (BioProject accession number PRJNA976177). All codes used in this manuscript is available on GiTHub (https://github.com/UiT-Tumorbiology/Mammary_EMT_Signature).

## Ethics statement

Ethical approval was not required for the studies on humans in accordance with the local legislation and institutional requirements because only commercially available established cell lines were used.

## Author contributions

EK and MP conceived and designed the study. EK and TF performed the experiments. EK, SS and MP analyzed and interpreted the data. SJ facilitated RNA-seq experiments. JL, TG, GM, and OMS provided the cell lines used in the study. EK, SS, and MP wrote the manuscript. All authors contributed to the article and approved the submitted version.

## Supplementary material

Supplementary Figure 1 Optimization of number of clusters. **(A, B)**, and **(C)**. Connectivity (left), Dunn index (middle), and silhouette width (right) for **(A)** the Cancer Cell Line Encyclopedia, **(B)** TCGA, and **(C)** METABRIC. Final number of clusters selected for this study are marked in red.

Supplementary Figure 2 Disease Specific Survival and Progression Free Interval. **(A)** and **(B)** Kaplan-Meier curve displaying the estimated Disease Specific Survival (DSS) **(A)** and Progression Free Interval (PFI) **(B)** up to twenty-five years for basal-like breast cancer patients clustered in the EMT-up cluster 1 or the partial-EMT cluster 5 in TCGA. **(C)** and **(D)** Kaplan-Meier curve displaying the estimated Disease Specific Survival (DSS) **(C)** and Progression Free Interval (PFI) **(D)** up to twenty-five years for basal-like breast cancer patients clustered in the EMT-up cluster 1 or the partial-EMT cluster 3 in METABRIC.

Supplementary Table 1 Complete expression matrix of all genes included in the analysis of the RNA-Sequencing data from the three mammary EMT models.

Supplementary Table 2 Complete list of enriched hallmarks identified by Gene Set Enrichment Analysis results from the three EMT models.

Supplementary Table 3 Complete list of genes found to be significantly differentially expressed in the Gene Set Enrichment Analysis within the hallmark Epithelial Mesenchymal Transition for each cell model.

Supplementary Table 4 List of all significantly differently expressed genes in all three EMT models included in the 265-gene mammary EMT signature.

Supplementary Table 5 Analysis of overlapping genes between the 265-gene mammary EMT signature and previously published EMT signatures from the EMTome database.

Supplementary Table 6 List of the 265-gene mammary EMT signature and their response in the EMT models and their gene clustering in the breast cancer cell lines and patient samples.

Supplementary Table 7 Complete list of enriched hallmarks identified by Gene Set Enrichment Analysis results from comparing basal-like breast cancers clustering either as EMT-up or partial-EMT in TCGA and Metabric.
