# Supplementary figures and images for: Identification of a core EMT signature that separates basal-like breast cancers into partial- and post-EMT subtypes
Source: Front Oncol. 2023 Dec 4;13:1249895. doi: 10.3389/fonc.2023.1249895 (PMC10726128; doi:10.3389/fonc.2023.1249895)

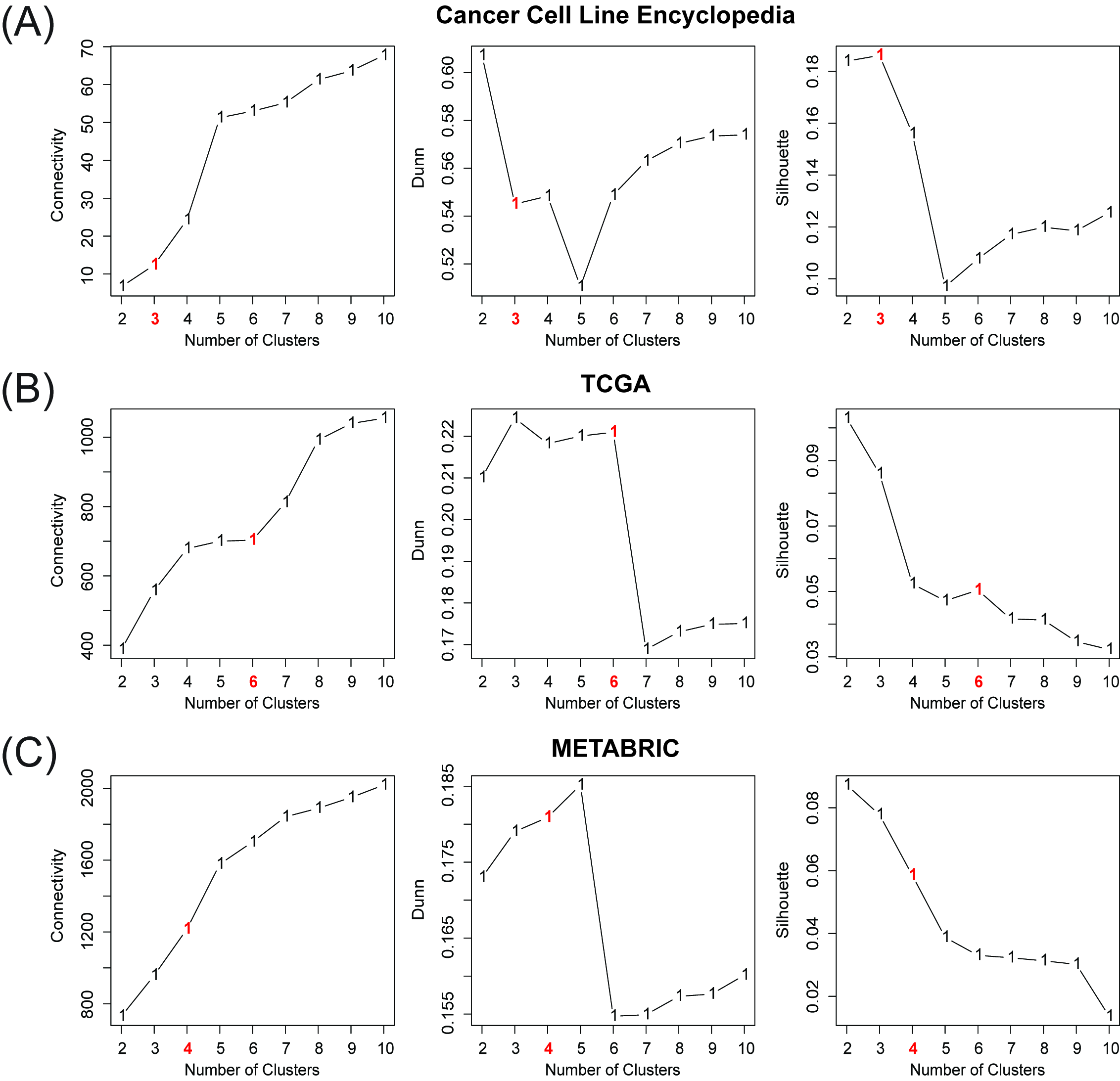

Supplement: Supplementary Figure 1 — Optimization of number of clusters. (A, B), and (C). Connectivity (left), Dunn index (middle), and silhouette width (right) for (A) the Cancer Cell Line Encyclopedia, (B) TCGA, and (C) METABRIC. Final number of clusters selected for this study are marked in red. [file Image_1.tif]

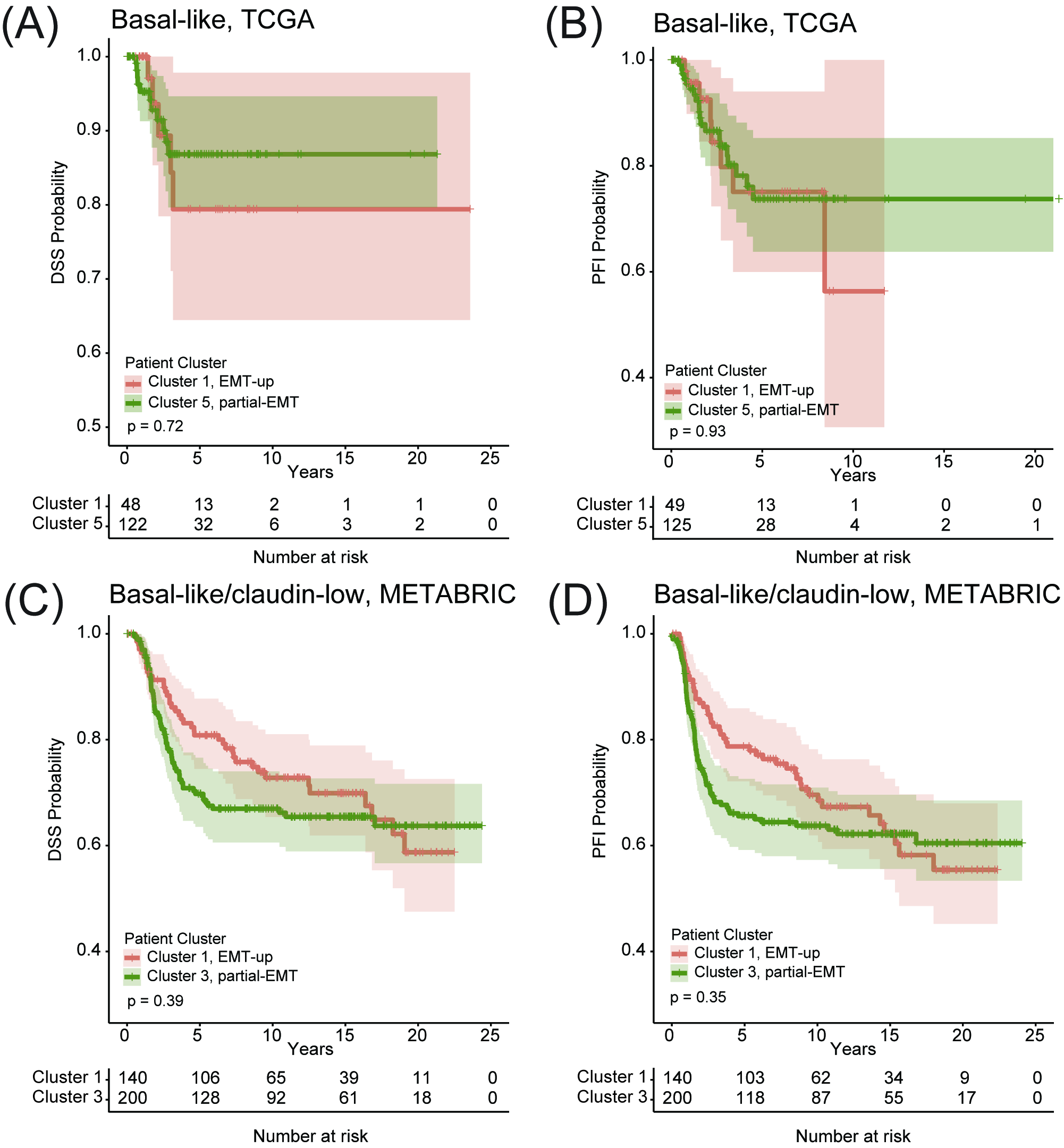

Supplement: Supplementary Figure 2 — Disease Specific Survival and Progression Free Interval. (A) and (B) Kaplan-Meier curve displaying the estimated Disease Specific Survival (DSS) (A) and Progression Free Interval (PFI) (B) up to twenty-five years for basal-like breast cancer patients clustered in the EMT-up cluster 1 or the partial-EMT cluster 5 in TCGA. (C) and (D) Kaplan-Meier curve displaying the estimated Disease Specific Survival (DSS) (C) and Progression Free Interval (PFI) (D) up to twenty-five years for basal-like breast cancer patients clustered in the EMT-up cluster 1 or the partial-EMT cluster 3 in METABRIC. [file Image_2.tif]
